# Commentary on, “Generation of Three-dimensional Human Neuronal Cultures: Application to Modeling CNS Viral Infections”

**Published:** 2019-03-28

**Authors:** Leonardo D’Aiuto, Nicholas Radio, Vishwajit L. Nimgaonkar

**Affiliations:** 1 Department of Psychiatry, University of Pittsburgh School of Medicine, Western Psychiatric Institute and Clinic, 3811 O’Hara Street, Pittsburgh, PA, US; 2 Thermo Fisher Scientific, Cellular Imaging and Analysis, 100 Technology Drive, Pittsburgh, PA, US

Human herpes encephalitis (HSE) is the most devastating consequence of Herpes simplex virus type 1 (HSV-1) infection of the central nervous system (CNS). The mortality rate of untreated HSE is approximately 70%^1,2^. With the advent of acyclovir (ACV), a highly effective antiviral against lytic HSV-1 infections, the mortality rate has decreased to approximately 20%^3^. However, the majority of encephalitis survivors suffer from permanent sequelae such as severe neurologic deficits and impairment of anterograde and retrograde memory, executive functioning and language^4^.

Besides HSE, an increasing array of evidence suggests the involvement of HSV-1 in the etiology of Alzheimer’s disease (AD)^5^. *In vitro* studies show that ACV treatment can reduce the accumulation of β-amyloid and phosphorylated tau proteins described in AD pathology, indicating that antiviral treatments may reduce the progression of the disease^6^. Despite the tremendous impact of ACV on the prophylaxis and treatment of herpes infections, HSV-1 resistance to ACV and its derivatives is being documented increasingly, particularly among immunocompromised individuals^7,8,9,10^. Furthermore, neurotoxicity has been repeatedly described as a side effect of ACV treatment in patients with renal failure^11,12^. Hence, the development of a new generation of potent, well tolerated anti-herpetic drugs with a mechanism of action different from ACV is indicated.

The identification of promising lead compounds in drug discovery is largely influenced by the cellular platforms^13^. Besides the choices of cell types to employ in a drug screening campaign, an additional inquiry has become increasingly relevant over the last few years: ‘2D or not 2D’? In other words, are two-dimensional cell culture systems sufficient for drug screening platforms? This Shakespearian paraphrase highlights new challenges that the field of drug discovery are facing regarding the generation of cellular platforms with higher predictive values of the drugs’ activity *in vivo*. Drug screening campaigns aim to make the workflow easy, fast and cost effective. These objectives are not particularly challenging when the test platforms in drug screening employ monolayer two-dimensional (2D) cell cultures. However, these practical and economical demands overshadow the most important prerequisites for a potentially successful drug screening campaign; i.e., a physiologically relevant culture system composed of disease-relevant cell types. Are 2D cultures a good proxy for a tissue environment?

Approximately 85% drugs fail during early clinical trials^14^. A portion of the inefficiency can be attributed to the inadequate representation of human tissue environments in 2D cultures^15^. Indeed, cellular responses in 2D monolayer cultures differ from their *in vivo* analogs. There is accumulating evidence showing that the drug response may differ significantly when cells are cultured as a monolayer or as a three-dimensional (3D) format^16,17^. Another important reason for drugs failing before moving to clinical trials (besides the species-specificity, organ-specificity and tissue specificity of the drug’s toxicity^15,18,19^) is the fact that cell phenotypes in cell-based toxicity assays measured in 2D monolayer cultures are not equivalent to their counterparts’ *in vivo*-tissue. Similar differences are even evident when comparing the drug response of cells cultured as 2D monolayer or 3D cellular aggregates. The latter is a better predictor of drug response *in vivo*^16,20^. Ongoing research in infectious diseases suggests that evaluating cellular functions in 3D culture systems may bridge the gap between *in vitro* and *in vivo* models of pathogen-host interactions^21^.

Different strategies have been developed to generate 3D cultures. Some of these approaches involve the use of scaffolds made from synthetic or natural materials, whilst others are based on the ability of specific cell types to self-aggregate and generate multi cellular 3D structures. The scalability, degree of effort, and costs of materials and reagents make these technologies difficult to adapt to high-throughput drug screening. An additional layer of difficulty comes with the high content imaging of 3D cell cultures.

The article “Generation of three-dimensional human neuronal cultures: application to modeling CNS viral infections”^22^ describes a prototype of scaffold-free adherent 3D (A-3D) cultures of central nervous system (CNS) cells in 96-well plate generated from human induced pluripotent stem cells (hiPSCs). hiPSC-derived neuronal cells can mimic the functionality of human CNS neurons, and can be generated in quantities compatible with drug screening workflows. The A-3D cultures consist of multilayered cell aggregates, whose thickness ranges from approximately 25 μm to 60 μm. These A-3D cultures show comparable size, as well as an acceptable well-to-well variability, which are important prerequisites for drug screening. Immunohistochemistry analysis showed features of a developing cortex in A-3D cultures. Importantly, these A-3D cultures are sustained by the extracellular matrix (ECM) generated by the differentiating cells. Considering the influence of the ECM composition on drug response and its unique composition in the brain^23–25^, the abundant secretion of ECM by the CNS cells represents another important advantage of the A-3D culture system.

A major problem related to the high content screening (HCS) of 3D culture platforms for drug screening is the rapid acquisition of z-stacks from multicellular structures. Historically, this has been conducted utilizing manual confocal imaging methods. More recently, there has been significant advancements in HCS methods that involve laser powered confocal instrumentation amendable to high throughput assays. The Cellinsight CX7 LZR instrument from Thermo Fisher Scientific is a 7-laser Nipkow Spinning Disc platform that has been optimized using fluorescent dyes conducive for spheroid phenotypic evaluations. The multiple pinhole Nipkow spinning disk confocal technology integrated into the optical path provides high-resolution imaging of thick samples. To ensure high throughput processing capabilities, the CX7 LZR features solid state laser-based illumination and is configured for fluorescent imaging in the UV through near-IR range.

To investigate the suitability of the A-3D culture system for high-throughput screening, we tested the inhibitory activity of increasing concentration of ACV, from 0.1 μM to 50 μM, on cells infected with a HSV-1 construct carrying EGFP and RFP reporter genes under the control of the viral promoters ICP0 and glycoprotein C (gC), respectively^26^. Uninfected and infected cells from two sets of experiments were analyzed using flow cytometry (FC) and the CX7 LZR High-Content Screening (HCS) platform. The half maximal inhibitory concentration (IC50) of acyclovir estimated by FC and HCS were comparable (3.144 μM and 3.121 μM, respectively), indicating the accuracy of the CX7 HCS platform for high-content measurements of 3D culture systems. The CX7 LZR was able to conduct the plate analysis including confocal Z-stacking in under 1 hour, whereas the FC method required approximately 4 hours to complete. Furthermore, use of the CX7 HCS platform overcomes the problem of cell damage caused by the proteolytic enzymes during the cells dissociation required for the FC.

In summary, the A-3D culture system paired with CX7 HCS technology paves the way for robust, rapid, and accurate high-throughput drug screening for inhibitors of neurotropic viruses that invade the human CNS.
